# Concerted Model of Healthcare for Awá Indigenous of Nariño, Colombia

**DOI:** 10.3390/ijerph191912250

**Published:** 2022-09-27

**Authors:** Harold Mauricio Casas Cruz, Blanca Estela Pelcastre-Villafuerte, Luz Arenas-Monreal, Myriam Ruiz-Rodríguez

**Affiliations:** 1 Faculty of Health Sciences, Medicine Program, University of Nariño, Pasto 52001, Colombia; 2 Centre for Health Systems Research, National Institute of Public Health, Cuernavaca 62100, Mexico; 3 Department of Public Health, Medicine School, Universidad Industrial de Santander-UIS, Bucaramanga 680002, Colombia

**Keywords:** Indigenous, healthcare model, primary health care, social determinants of health, community-based participatory action research, Colombia

## Abstract

Indigenous communities in Colombia are facing a critical health situation; alternative health care models based on the vision of the communities themselves are needed. The objective of this research was to create a health care model that decreases health inequities for the Indigenous Awá population of Nariño, Colombia. This study was guided by the paradigm of community-based participatory action research; the process was carried out in 2015 and 2016. The proposed Intercultural Health Care Model is essentially based on health promotion, disease prevention, community empowerment, social participation in health, decentralized health care and coordination between the two medicines (traditional and allopathic). Strategies such as those reported herein, with concerted efforts rather than imposition, maintain human rights and respect for the sovereignty and autonomy of Indigenous people.

## 1. Introduction

Indigenous communities in Colombia are facing a critical health situation, as demonstrated by indicators of high maternal, perinatal and childhood mortality, tropical diseases, malnutrition and tuberculosis, that are all significantly greater than those of the rest of the population. Meanwhile, the coverage of health programs is notably deficient, including the Expanded Immunizations Program (PAI in Spanish), institutional services for childbirth and prenatal care and basic health coverage [1].

Given that these communities are spread out across rural areas that are difficult to access, they face geographic, social and administrative barriers to effective access to health services. These barriers also include the location of Indigenous territories in areas of armed conflict, illegal crops, the presence of minefields and the environmental effects of illegal mining and voracious deforestation, which affect the autonomy and food security of this population [2] and result in isolation and exclusion from health, education and communication services, among others. This presents a difficult short- and medium-term situation for Indigenous communities, especially for the Awá Indigenous population in Nariño, which urgently needs attention to safeguard the continuation of their physical and cultural existence as determined by Colombia’s Constitutional Court [3].

Colombia’s health care model is characterized by health insurance, government assistance and hospital services in urban centers and populated areas, with a special focus on disease and emerging advances in primary health care (PHC) and social determinants of health (SDH). This model is not compatible with nor does it address the particular sociocultural conditions and health needs of Indigenous communities, and thereby it creates inequities.

The Awá population of Nariño is one of the most threatened ancestral populations in Latin America today, with health indicators confirming high maternal, neonatal, infant and childhood mortality and morbimortality from malnutrition and diarrheal and respiratory diseases that particularly affect children, pregnant women and older adults [4]. Further, while constitutional rights, international agreements and stipulations by Colombia’s Constitutional Court afford the members of these communities the status of “special protection” by the Colombian government, which is required by all areas of Colombia’s government, this is not fully being met.

The present study was conducted according to the framework of the Safeguard Plan for the Awá population, mandated by the Constitutional Court. This plan responds to the need and request by the Ricaurte civil authorities, traditional authorities and the Awá community’s Camawari Organization for the first author to define a health care model that incorporates interculturality as a central axis and that includes the theoretical principles of PHC and the SDH approach in the definition of an integrated health care model that effectively contributes to decreasing current health inequities and the barriers to access to services. The model is aimed at fostering dialogue and intercultural coordination between Western and traditional Awá medicines, and at providing intercultural, timely and quality health care with the resulting improvement in the health conditions, lives and physical and cultural continuity of the Awá population. This article describes the process for the concerted development of that model.

## 2. Materials and Methods

### 2.1. Design

This study was guided by the paradigm of community-based participatory action research (CBPAR) [5], which involves recognizing the members of the Awá community as social actors who are active participants in the research process. This approach also treats the researcher as a facilitator who guides and develops the process with the participation of the community. It is based on critical ethnography [6], which involves the insertion of the main researcher in the field. The process was carried out in 2015 and 2016 and was cyclical following the proposed methodology.

### 2.2. Participants

The participants in the research process were members of the Awá community in the municipality of Ricaurte Nariño, Colombia, and were over 18 years of age. They included youth, older adults, pregnant women and mothers in the community; Indigenous members of the Indigenous communal territories, where the Awá communal territories in Ricaurte Nariño are located; traditional health agents, traditional physicians, midwives, “sopladores” (within traditional medicine, these people blow people sick with fright) and traditional bonesetters; rural health promoters; health care users in the communal territories; community leaders, traditional authorities, principal and deputy governors, Indigenous council attorneys, sheriffs, members of the Indigenous guard and wise elders; among others.

### 2.3. Data Collection Techniques

#### 2.3.1. Analysis of Documents

Various documents were analyzed, including the Awá Community Life Plan—Camawari Organization, the 2012–2015 Ricaurte Nariño Municipal Development Plan, documents from the 2013 Health Information Analysis (ASIS in Spanish) of the municipality of Ricaurte Nariño, the 2014 ASIS of the Department of Nariño, the 2014 Journal of Health Indicators by the Nariño Department of Health, the 2014 Colombia ASIS, technical documents on Renewal of Primary Health Care (PHC-R) from the Pan American Health Organization, information about social determinants of health from the World Health Organization, and unpublished documents by the Camawari Organization on the health culture of the Awá, among others.

#### 2.3.2. Participant Observation

Participant observation took place throughout the research process over the nearly two years that the main researcher was immersed in the Awá territory in the piedmont coastal jungles in the region of Nariño, Colombia, to critically examine the theoretical concepts and anchor them in concrete realities [7]. The information was recorded in a field journal.

#### 2.3.3. Interviews with Key Informants

Eighteen in-depth interviews were conducted with key actors (Table 1) to delve further into the cultural meanings that the Awá people attribute to their health and disease processes, as well as to further explore their perceptions of health services, social determinants of health and their general worldview of life and health.

The criteria for defining the number of participants interviewed were designed to be representative of the Awá communal territories to ensure the participation of at least one Indigenous person from each of those territories, particularly those located in the most remote territories. Representation was also sought from each group of traditional agents, women and Awá leaders. The traditional authorities were responsible for directly designating each interviewee, which contributed to the endorsement and legitimization of the testimonies.

The interviews were extensive and in-depth: at least 14 interviews were conducted over 8 non-consecutive days, on different days of the week and times of day. Some were even conducted at night because of ritual exercises in which traditional agents participated; the rest were conducted in fewer than 8 days but still took more than one session. Interpreters were used to translate the questions and the conversations. The number of participants was determined by the Camawari Organization. The outline for interview can be consulted as supplementary material.

#### 2.3.4. Focus Groups

Sixteen focus groups were created, and representation from the entire Awá territory in the municipality of Ricaurte Nariño was ensured. Each group had an average of 10 members and included pregnant women, traditional health agents (traditional physicians, midwives, “sopladores” massage therapists, “pulsadores” (within traditional medicine these are people who can “see” through the pulse; by pressing, the sick “blood speaks”), bonesetters, and rural health promoters), and community leaders and traditional authorities (principal and deputy governors, Indigenous council attorneys, sheriffs, members of the Indigenous guard, and wise elders, among others).

The focus groups were aimed at exploring the Awá worldview about health and their perceptions related to the Colombian health system and the care provided by health institutions, as well as elements of thought that constitute social determinants of health for the community.

Colombia’s Ministry of Health and Social Protection financed travel, lodging, food and logistics for the participants. Health care was provided to the population after the research activities were conducted, including weight and size measurements, medical consultations, medication administration, rapid tests for malaria, samples for tuberculosis and health education.

The discussions were moderated with help from Camawari Organization team members, Indigenous government leaders, Indigenous health promoters in each communal territory and teachers from the rural schools in some of the communal territories. Bilingual Indigenous people were present in each group and facilitated translation of the conversations. The outline for focus groups can be consulted as supplementary material.

#### 2.3.5. Group Discussion

Upon completion of the focus groups, the Indigenous government leaders and the Camawari Organization designated the key participants for the seven discussion groups (representatives from the communal territories where there are health centers and from strategic regions where the other communal territories are located).

The discussion groups focused on analysis, debate and the rationale regarding concepts of PHC, the key elements of the Life Plan, the worldview of health and life, traditional Awá medicine, their social determinants and their perceptions of health services that should be incorporated in a health care model. Technical elements were facilitated for the participatory and concerted development of the Intercultural Health Care Model.

In addition to Awá community members, the discussion groups also included officials from the Ricaurte local hospital, the local health department, traditional authorities, and leaders from the Camawari Organization.

### 2.4. Procedure

**Figure 1:**
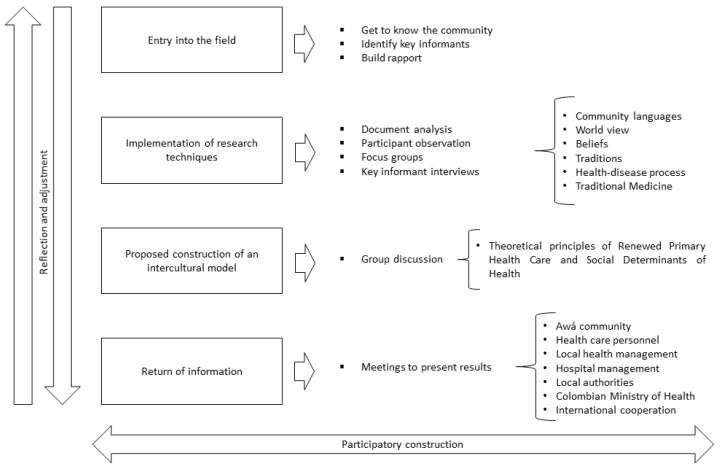
Research procedure.

This investigation was carried out over four cyclical stages, each of which complemented the others. The stages adhered to the mandatory process of pre-consultation and consultation, which is a right for the country’s ethnic groups. The following figure summarizes the stages (Figure 1).

### 2.5. Analysis of the Information

Since a large amount of the information that was obtained through the interviews and focus groups was in the Awapit language, it had to be translated to Spanish by a bilingual Awá person. The researcher recorded the interviews with the support of an Awá group belonging to the Camawari Organization. They were bilingual (Spanish and Awapit, the native Awá language) and worked in the organization’s health area as community health agents in the Awá territory. The Camawari Organization’s health team later transcribed the interviews, which as the official voice of the Awá people, ensured that the texts were the voice and the official version of the conversations and the respective translations.

For qualitative analysis, the information obtained from all the techniques was organized into categories using a data matrix with inductive or emergent categories. This information was compared with the observations from the field journal and used as feedback. Propositions that synthesized the results were generated by triangulating the information from the different techniques.

Participant observation, interviews with key informants and discussion groups made it possible to delve further into the information obtained from the focus groups and explore concepts of health and illness among the Awá population.

### 2.6. Ethical Considerations

The present research was approved (5 September 2014, code of approval IC: 708) by the Ethics Committee of Mexico’s National Institute of Public Health, for which the main author received her doctorate and where this investigation served as her thesis project. The study posed no risks to the participants; it complied with pertinent national laws and was guided by ethical principles for working with humans and ethnic groups. Under the traditions, social organization and policies of the Awá of Ricaurte, and in compliance with the collective rights and special legal status of Indigenous populations in Colombia, who are considered “subjects of collective rights”, the main researcher provided the traditional Awá authorities with the sufficient required information about objectives, mechanisms, techniques, the contributions of the investigation and the concerted development of the model through free, autonomous and confidential decision-making, as indicated in the general informed consent.

During the Awá general assembly held in late 2014 in the communal territory of Vegas, the government of the Awá territory and the community assembly provided their collective informed consent [8], endorsement and full permission to proceed with the process. The collective informed consent process was facilitated by the Camawari Organization on behalf of the government leaders as the highest authorities of the Awá councils. In addition, oral informed consent was obtained from all individual participants included in the study.

The final version of the research was submitted for validation and approval by the Awá community through a process that had been defined by this ancestral people. This involved presenting the entire research process and results to the governors of all the Awá people who were associated with the Camawari Organization, followed by a presentation and discussion at a minimum of two large community assemblies. The final version of the texts and the presentation of the research were endorsed by the Awá people in late 2015.

## 3. Results

The results presented herein focus on the concerted development of the model.

### 3.1. General Context of the Awá Community

#### 3.1.1. Geographic Location

The Awá Community’s geographic location is difficult to access. The main communication route for the municipality is a road that runs along the Colombian Pacific from Pasto to the Port of Tumaco. The other routes are secondary and tertiary roads that have few kilometers that are suitable for vehicular transit, in addition to horse and pedestrian paths that require 18 to 24 h of walking to reach the most remote communal territories.

#### 3.1.2. Demographic Aspects and Social Organization

Roughly 90% of the population of the municipality of Ricaurte is Awá, and the specific statistics about this community are nonexistent. Given the significant percentage of the population of the municipality that is of Awá ethnicity, this study attributed the official Ricaurte figures to the Awá community. The demographics in Ricaurte Nariño showed a significant reduction in the 0 to 4- and 5 to 9-year-old age groups [4], which was likely related to the high degree of neonatal, infant and childhood mortality.

The Awá population in Ricaurte Nariño is organized into 14 communal territories, with a population of roughly 15,000 Awá [9] residing primarily (85%) in the rural area of the municipality. The communal territories are governed by Indigenous councils, which represent the highest authority in these lands [10]. The councils are made up of the principal or senior governor, a deputy governor, a council attorney, a secretary, a treasurer, several sheriffs and a mayor. All of these positions are elected for a term of one year at community assemblies in which the entire community is required to meet for several days to select their authorities, who are legitimated through this democratic process.

The Camawari Organization’s primary functions are legal and administrative representation of the councils, management of projects, programs and life plans for the communal territories, preservation of ancestral rights and systematic pursuit of the security and physical, cultural and material protection of the Awá territory as a whole.

### 3.2. The “Katsa Su”, the Awá’s Concept of Land

**Figure 2:**
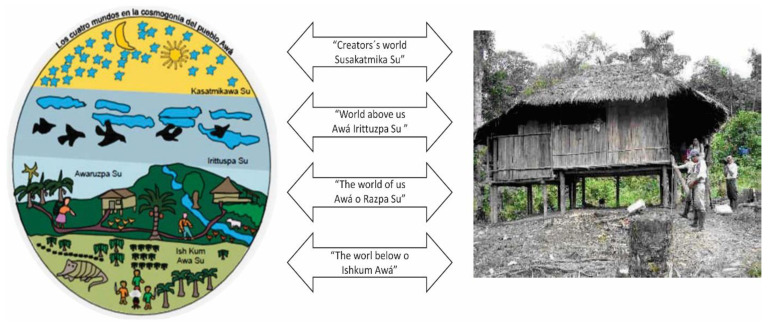
The “four worlds” of the Awá. (Source: Plan de Salvaguarda Étnico Awá, 2009. Photograph of a typical Awá house, courtesy of Camawari, 2010).

For the Awá, who refer to themselves as “Inkal Awá” or “children of the mountain”, the concept of land goes beyond the material concept of the land, earth or geographic areas that they inhabit. Their thinking and worldview about life and health, their social expressions, uses, customs, beliefs and cultural practices related to life and health, and even the layout of the typical Awá house—Yat Awá—revolve around the belief in the existence of “four worlds” (see Figure 2). Further immersed in their conception of land is a set of collective and individual rights that both they and others must respect, encourage and embrace. For the Awá, the land is even more important than individual rights and the very life of each Indigenous person.

Awá families are widely spread out across the communal territories to ensure that considerable distance is maintained among homes. This custom specifically relates to their cultural conception of the land, in which large physical and spiritual spaces are needed in their worldview of the “four worlds”. Generally, various family members live in one home, including grandparents, parents, children and partners. Over time, the older children form their own families and become independent of their parents.

### 3.3. Social Determinants of Health (SDH) for the Awá

The most important elements in the Awá vision of SDH are:

(1) The land: the central focus of their worldview of life and health is ancestral land, which is “material and spiritual” and supports their beliefs, uses and customs, particularly the belief in the existence of the “four physical and spiritual worlds” that govern their behavior and social and cultural relations and reflects a deep relationship with nature. For the Awá, the supra material conception of land has its equivalence and representation in the construction and layout of their homes.
(2) The mountains and jungles: the location of their sacred sites, which are inhabited by the spirits of their ancestors, reflecting the Awá’s intense cultural relationship with “mother earth.” The natural resources, forests, jungles and plants, as well as the wild animals that provide them sustenance and food security and sovereignty, are key to the Awá’s “Law of Origin”, which is based on the principle of “taking just what is needed from nature” to survive, without requiring more.
(3) Traditional medicine: with its beliefs, wisdom and ancestral knowledge and “the traditional physicians and their ongoing relationship with ancestral spirits”, which explain the uses, customs, behaviors and cultural practices related to health and make it possible to prevent, treat, purify and cure spiritual illnesses. In traditional medicine, a disease can be triggered by the influence of factors that are external to a person, such as plants or nature spirits, as well as by internal factors related to the spiritual dimension of the person. The latter is crucial to healing. Generally, it can be said that spiritual illnesses fall within culture-bound syndromes [11].
(4) Food security and sovereignty: the land permits and provides unlimited resources and sources of ancestral food, such that families freely moving through the mountains and jungles without restriction is guaranteed for planting, harvesting, hunting, fishing and eating for the security and physical preservation of the Awá.

It is worth noting that the Western health system was not mentioned at any time during the construction of the Awá perception of SDH and the corresponding agreements. Rather, there was a general sense that institutional health services were associated more with the disease process than with their conception of health.

### 3.4. Concerted Intercultural Health Care Model

Testimonies by the participants related to problems they have faced seeking care, as shown in Table 2, were the starting point for the reflection that led to the Concerted Model.

Proposal for Permanent Intrahospital and Extramural Health Care in Communal Territories.

The model begins with a series of proposals by the Awá of Ricaurte Nariño for intrahospital health care. These proposals essentially involve the need to redirect services and for mobile and multidisciplinary health teams to provide appropriate intercultural programs and ongoing extramural health care. They also include ongoing dialogue about the knowledge of traditional Awá medicine in the development and operations of an intercultural health care model in communal territories, one that is preventive, integrated, inclusive, participatory and oriented towards problem-solving.

Based on their experience, the Awá community proposed their SDH and problematics, as well as general guidelines for the approach and for progressive improvement through intersectorality and community participation. Their proposals are as follows:

- The creation of an Indigenous Health Services Provider Institution (IPSI in Spanish) to organize, administer and operate intercultural health care for the Ricaurte Awá community, with its administrative and service headquarters in the municipal capital and outpatient health care services offered by Western and traditional physicians. This proposal includes at least seven Mobile Health Teams (MHTs) made up of family physicians, dentists, nurses, nursing aides or public health workers, health promoters and environmental sanitation promoters working in coordination with traditional Awá medicine practitioners (prior meetings would ensure both a common language and shared goals). These units would be permanently and strategically located in seven communal territories throughout the Awá territory and be open 24 h/day, 7 days/week and 20 days/month (with a 10-day rest period). They would play a strong role in health promotion and disease prevention, in the search for and early detection of individual, family and community risk factors for health, in intervention in those factors and the provision of low-complexity extramural medical care.
- The MHTs would be trained and be able to solve problems. Their operations in the Awá territory would be logistically autonomous, and they would also handle medical and obstetric emergencies, or when necessary, stabilize and refer patients to second- or third-level complexity services without negatively affecting the health care that the local hospital should continue to provide, which would continue to be the referral hospital for primary care. At the same time, the Awá would continue to receive health services from traditional practitioners in close coordination with the MHTs. The Awá families would be the first link in the community’s participation in health, with activities related to self-care, community-based public health surveillance, referrals and counter-referrals of patients, basic sanitation, and health promotion and prevention.
- The progressive conversion of the Ricaurte hospital into a second-level hospital is proposed through the implementation of the human resources, technology and facilities needed to address not only general low-complexity medical needs but also medium-complexity cases. To this end, basic medical surgical specialties would be provided, including pediatrics, internal medicine, gynecology, obstetrics, general surgery and anesthesia to treat basic medical and surgical cases for children, women and adults, and thereby minimize referral of patients to other cities. Subsequently, the MHTs would be strengthened by family physicians who would provide needed medical care, thereby expanding and improving problem-solving capacity directly in the field and in the local hospital, and through this action preventing displacement of the Awá. This also prevents their feeling of being uprooted when they are referred from their territory to the local hospital or other institutions in the service network, as these spaces are very foreign to them and where, generally, they are far from their relatives and friends.
- The intercultural appropriateness of all institutional health programs at the local hospital and the institutions in the network is envisioned to be in accordance with the Awá community’s worldview of life and health. This process would be a product of intercultural dialogue on health—an “exchange of knowledge” that enables taking concerted actions that effectively impact the current health situation of the Awá population. The proposal also includes appropriate physical spaces, food and accommodations, as well as traditional physicians who offer health care in the health institutions and are dedicated to “spiritual” care and assisting Awá patients who require hospitalization. This would undoubtedly contribute to successfully treating cultural illnesses that are specific to the Awá population, or “spiritual and physical” illnesses.
- It is proposed that housing be made available in the municipal capital of Ricaurte for Indigenous patients who are referred to the local hospital from the most remote communal territories and for their families and companions so that they have a dignified place to stay and take meals during patient visits and hospitalizations. This would also be used by postpartum women to get the rest they need during at least their first few days on the diet that the Awá women are accustomed to eating after childbirth. This would mitigate the negative effects of the institution’s decision to discharge women the day after childbirth and avoid the medical and cultural implications of the mother and child taking a long trip back home, which often requires traveling for more than two days in difficult climate conditions and along the poor roads that are typical of the region.
- Ongoing support for traditional Awá medicine is proposed through the training of new traditional physicians and midwives by providing support or incentives for the work of traditional health agents through modalities such as “food for work” or “food for training” programs, which have been implemented in the past by international cooperation agencies in the region, such as the United Nations World Food Programme [12]. This type of support can also be applied for promoting community medicinal gardens.
- The participants proposed that the traditional and civil authorities in the municipality of Ricaurte, the department of Nariño, national authorities and international cooperation agencies work together to solve the serious problems in the Awá territory with the ongoing humanitarian situation and armed conflict, through urgent humanitarian demining of the territory, suspending current and future large-scale mining and forest exploitation, stopping aerial glyphosate spraying of mountains and jungles, taking concerted efforts to develop and implement options to replace and end the use of illegal crops, carrying out appropriate, adapted, concerted and sustainable community development projects that ensure food security and sovereignty in accordance with their uses and customs, implementing environmentally sustainable alternatives for supplying safe water, and appropriately disposing of excrement and solid and liquid wastes.
- Lastly, the Awá community proposed support and capacity-building for their traditional medicine agents, incentives for cultivating medicinal plants for the rituals, uses, practices and customs of their health culture, meetings and dialogues for the exchange of knowledge, and coordination with Western medicine, particularly with the proposed mobile teams.

### 3.5. Characteristics of the Model and Operational Strategies

**Figure 3:**
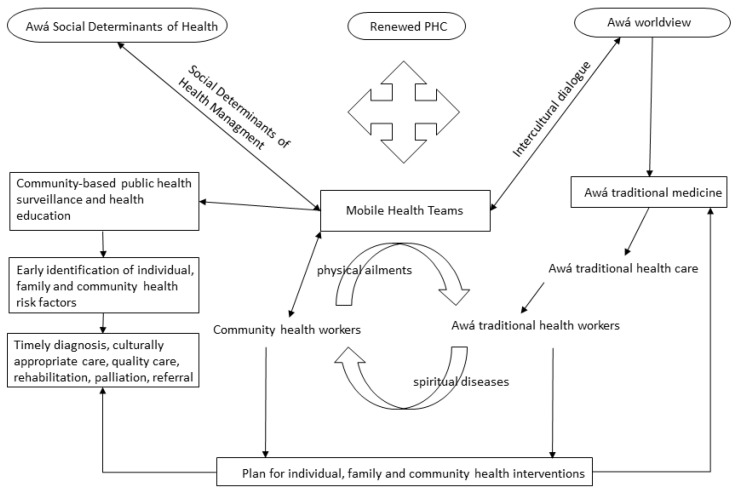
Concerted intercultural health care model. (Source: Authors’ own construction, based on the proposals of the qualitative field work and consultation with the Awá people).

(1) Concerted efforts through ongoing dialogue about the knowledge of Western and traditional Awá medicine; dialogue based on recognition, respect and mutual coordination.
(2) Community participation in health through the social mobilization of Awá families and communities, intercultural adaptation of health education on self-care and disease prevention, community-based public health surveillance, and establishing an intercultural dialogue between Western and traditional medicine.
(3) Intersectorality: Institutional, sectoral, intersectoral, community and cross-sectoral coordination for an integrated SDH approach.
(4) Reorienting institutional health services according to the social, cultural and geographic needs of the municipality and the Awá territory.
(5) Intercultural appropriateness of institutional health programs and services through ongoing intercultural dialogue between the two medicines and health cultures.
(6) Ongoing education for Mobile Health Teams and the community, and capacity-building, training and exchange of knowledge for the institutions and the community. This includes training Awá people as health technicians, technologists and professionals for subsequent community service and ownership of their health processes.
(7) Health sector advocacy of programs, projects, plans and policies that lead to improving the health situation of the Awá population.
(8) Strengthening institutions to expand coverage, services, technologies and facilities in the service network, including telecommunication, communication and information technologies for improving the problem-solving capacity of the MHTs in Awá territories.
(9) Ongoing planning and evaluation for organizing services, managing health risks, providing quality care, ensuring the sustainability of the model, accomplishing concrete processes, achieving health impact goals, improving the quality of health and consequently contributing to the physical and cultural preservation of the Awá population in Ricaurte Nariño, Colombia. The integrated model can be seen in Figure 3.

## 4. Discussion

The results of this study establish the basis for an intercultural health model that is focused on primary health care and the social determinants of health from the perspective of the Awá worldview. This model integrates ancestral medicine into the Colombian healthcare model. The Indigenous people accept the country’s health system—they do not reject the services, but they are clear about the differences in approaches between their diseases that they believe can only be solved by traditional agents and the diseases from outside that Western doctors can address. What the model presents is a way to build bridges that have not yet existed between the two health systems, the lack of which has contributed to the current health status of the Awá and other ancestral people in Colombia, with clearly greater preventable morbidity and mortality, precisely due to the cultural break that has existed, among other reasons.

Thus, the model that the Awá community has built is itself an integration of the two health systems. Corso et al. point out that leadership and participation of Indigenous populations in health systems is fundamental to integrating their healing practices and the traditional Indigenous model of care within the Canadian health system [13]. The Awá people defined their version of both SDH and Renewed PHC. They defined the components one-by-one, beginning with the door used to enter the health system. In the Western version of the Colombian health model, people enter through the emergency department, outpatient visits, growth and development consults, prenatal care visits, etc. Meanwhile, for the proposed Awá model, the entryway is through their own use and customs, their traditional health agents, and their health programs. As the model indicates, the Western world should culturally adapt and adjust to this. For example, community-based public health surveillance is based on recognizing, valuing, participating in and the functioning of traditional health systems with mobile teams. This should function effectively and collaboratively. Traditional systems (with their health agents) can collaborate in the characterization of their territory, in the early identification and intervention of risk factors and in a positive SDH intervention. For the Awá version, this means that the Western world truly sees and includes health in all policies, which, in the case of Colombia, involves transcending the service- and curative-based model that has not only served as a foundation but has also been the cause of its relative failure in the area of public health.

Thus, each component of the health care model proposed by the Awá community is similar to the elements in Renewing Primary Health Care [14]: namely, point of entry, first contact, family and community orientation, health promotion and disease prevention, and social participation, among others. Its logic is territorial rather than contractual, and it is supported by participatory planning of the work in Awá communal territories, the coordination of the MHTs and their interventions, community participation, coordination and cooperation between the two medicines (traditional and allopathic), and an administrative component that guarantees the financial sustainability of the model. Several studies report the importance of the participation of Indigenous populations in the identification of needs and the specific orientation of the health system they require [13,15].

To ensure this coordinated work, there could be the integration of what other models have called “intercultural bridges”, or Indigenous health agents. These are Indigenous men and women whose primary function is to serve as liaisons between the people in the community, or in this case between traditional doctors and health services staff. Ideally, these Indigenous agents must be bilingual and professionally trained in the area of health as doctors and nurses, and as such, they are also bicultural; that is, they understand and share the Indigenous population’s worldview—including their traditional medical knowledge and practices—and they understand the organization and functioning of the institutional health system to which they also belong [16]. A systematic review that aimed to identify the characteristics of Indigenous models of primary health care found that the incorporation of culture is fundamental, which makes sense from this perspective of “cross-cultural bridges” [17].

This model is the result of an in-depth process of reflection on their health situation and their perception of institutional health care, and the participation and concerted efforts of the Awá community. This health model is intercultural, inclusive, preventive, and integrated, and it addresses the complex problem posed by the SDH. In some Latin American countries there are policies and strategies to incorporate the intercultural approach and Indigenous medicine with the biomedical model within the health care systems, although not all countries have had the active participation of Indigenous peoples, as was the case with the Awá community [18,19,20].

Strategies such as those reported herein that use social participation with concerted efforts rather than imposition revolve around the framework of human rights and respect for the sovereignty and autonomy of Indigenous people, as expressed in various international documents [14,21,22]. As other authors have pointed out, the result is the strengthening of the capacity of communities based on their own view and regardless of models outside of their worldview, while still retaining elements and resources from existing services. This constitutes a key element for reducing health inequities [23,24,25].

The concerted development of the model came to fruition through political and administrative advocacy for local, regional and national authorities to incorporate it in their respective development plans in such a way as to ensure its implementation and financial, social and cultural sustainability. It was incorporated in the 2016–2019 Ricaurte Nariño Municipal Development Plan, and its incorporation in the 2016–2019 Department and Territory Health Development Plan was studied but not carried out. Another type of agreement with the government was probably required after the model was agreed upon. Gibson et al. synthesize from a literature review that in the design and planning phases of primary care interventions, securing funding requires multiple government funding arrangements. These arrangements between Indigenous community-controlled health services and governments tend to be more complex than those between governments and conventional services [26]. These funding challenges have also been described by other authors who reported the complexity for Indigenous people of coordinating multiple funding sources [27].

By the time this study was completed and the intercultural health model was constructed (2016), the national government in Colombia was already operating in the department and municipality of Nariño with its earlier development plans. The mandates “Colombia 2018–2022”, the “Pact for Colombia National Development Plan” and the “Pact for Equity”, as well as the public health priorities of Colombia’s Ministry of Health and Social Protection, state that Colombia should make advances in the construction of intercultural health models and health models based on PHC and SDH for Indigenous people with the necessary intercultural adaptations.

The 2020–2023 Nariño Departmental Development plan and the Ricaurte Municipal Development Plan express a commitment to including the model in the differential management of vulnerable populations. Thus, the model met one of its most important aims, which was to influence administrative policies to include the model in their development plans. Nevertheless, this accomplishment will materialize when the country makes progress on building the Intercultural Health System of the Indigenous People (SISPI in Spanish), which serves as the legal, political and even financial administrative framework for providing the support and sustainability needed for full implementation. In other words, to have an impact and to see health results, any model of this nature needs local and national support and management so that it has sufficient human and financial resources.

## 5. Conclusions

For the first time in the region, an Indigenous community met over months in its own territory to reflect, discuss, propose and participate in concerted efforts to develop a health care model that recognizes and respects their worldview of health and life and incorporates it into decentralized individual, family and community health care. It was an important participation exercise. This concertation exercise reflects the achievements of the Indigenous people of Cauca in terms of the struggles they have encountered in their own determination of a health system oriented towards good living, ancestral wisdom and collective political action [28]. The result is a model that should significantly contribute to improving the current health situation of the Awá population. There are no records in the country of this process of collective construction.

The model is essentially based on health promotion, disease prevention, community empowerment, social participation in health, decentralized health care and coordination between the two medicines. Its implementation requires the coordination of all actors in Colombia’s health system, including the Ministry of Health, the Interior Ministry and the Ministry of Defense (to solve the complex territorial, humanitarian and security problems described), and the ministries of Housing and Potable Water, Agriculture, Education and Communications; other national and regional ministries and entities such as the local health department, the Ricaurte municipal hospital, insurers, complementary service networks, the department’s health institute, and the Nariño government; and other entities that are involved in and responsible for actions related to the SDH of the Awá community and the successful financing and operations of Mobile Health Teams and the model in general.

In addition, traditional health care, social participation by the Awá community in self-care, active participation in community-based public health surveillance and international cooperation agencies in the region should join together to complement the actions and interventions of their respective responsibilities. This needed intersectoral coordination, which is emphasized in the theoretical principles of PHC and SDH, does not generally take place effectively in Colombia. Only in this way will what is stipulated in legislation on health and the rights of Indigenous people in Colombia be put into practice. Further, only in this way can the drive towards the extermination of this Indigenous community be stopped.

## 6. Strengths and Limitations

This operative research was the first approach to the concerted construction of an intercultural health care model for Colombia based on the theoretical foundations of Primary Health Care with a focus on Social Determinants of Health and in coherence with the worldview of the Awá Indigenous people; up to this moment in Colombia and despite the normative advances on the subject, there has been no significant development in PHC or in SHD; the contribution to the public health of the country is of great relevance, pertinence and high impact for the health of Indigenous peoples, some of them, as in the case of the Awá people, in a clear process towards physical and cultural extermination.

The research provides elements for the implementation of other health care models based on PHC/HSD in different regions of the country and proposes a methodological route to accomplish this goal, with the mechanisms for community participation and the steps to achieve it, which is unprecedented in the country.

One aspect that eventually limits the progress and scope of this project is economic resources; so it is expected that its implementation and support will be so convincing that national, regional and local health authorities and international cooperation agencies will be interested in financing the proposed model.

In the Colombian context, achieving the development of the concerted model faces a structural obstacle that has to do with the neoliberal and market model on which the Colombian health system is based, and, as discussed by some authors [28], the concerted Indigenous health model, if implemented, runs the risk of being subordinated to this neoliberal model that is not sensitive to the needs and characteristics of the population and has a focus on economic profitability.

Another limitation that becomes a threat to the implementation of the model is the humanitarian situation and the public order problems that the region has historically experienced, which coincides with the map of settlements of the Awá people and would eventually become a clear limitation for the process. Given this situation, it is hoped that the peace negotiation process that the Colombian government is carrying out will contribute to reduction of armed conflict and will allow this and other initiatives for the care of ethnic groups and for vulnerable groups in general in the country, particularly for the Awá Indigenous people, to prosper for their benefit.

## Figures and Tables

**Table 1:** Interviews with Key Informants.

Role	Number	Sex (Female/Male)	Age	Communal Territory	Distance to Municipal Capital (Walking)
Traditional authorities: governors, other members of councils	5	5 M	42–65	Pialapí, Nulpes Medio, Alto, Pueblo Viejo, Gualcalá, Vegas	12–24 h
Midwives (males)	3	3 M	34–58	Nulpes, Pueblo Viejo	18 h
Healers	3	1 F, 2 M	45–62	Gualcalá	24 h
Pregnant women	2	2 F	23, 35	Nulpes	18 h
Wise elder	1	1 M	88	Pueblo Viejo	14 h
Leaders and representatives	2	2 M	33, 42	Vegas, Integrado M	8–12 h
Other Indigenous from communal territories	2	1 F, 1 M	27, 53	Guaduales; Milagrosa	18 h

Source: Developed by the authors based on attendance record.

**Table 2:** Testimonies from participants about institutional health care and the model.

“[Awá Women] Feel a Lack of Trust, Embarrassment When Being Examined, Looked at or Touched by Western Physicians” “the Food They Give [in the] Hospital Is Not Good, Upset the Stomach, They Don’t Like It (Midwife from the Pueblo Viejo Communal Territory)
“[to get health care we have to] stay up until four in the morning and stand in line to get a number”
“it takes several days for someone who is sick to be treated [in the] hospital”
“the hospital discharges Awá women who recently give birth the day after the birth, so they go home, regardless of their having to walk many hours on foot after childbirth”
“medical attention at the Ricaurte hospital in the communal territories is at most a one-week visit or less per year”
“…we need a group of doctors or a health team that rotates from here to there, that visits the Awá families weekly, sees the mom, the dad, the children, sees what their housing is like, the water, that comes back again and visits all the three hundred plus families in this communal territory and is permanently around the community. But this health group should of course also include the traditional physicians from here, where the midwife can provide care, other Awá traditional doctors of ours, be made up of Western medicine with a doctor, nurse, health technician, sanitation, and have functions in the community, so if tomorrow a health problem presents itself we find that health team in the communal territory, they provide us with medical care and also with a traditional physician and in that way the Awá family begins to be protected, to be cared for by a health team, given guidance, vaccinations, prenatal care if the father gives his permission, examine someone who is coughing, collect phlegm for tuberculosis, that there be health care like we have never had in the communal territories, but health care through our concerted efforts, then the community understands that what we are going to do now is see how health care is going to be for us Awá” (interview with a member of the communal territory)

Source: Developed by the authors based on interviews.

## Data Availability

Data can be made available upon request.

## Supplementary Materials

The following supporting information can be downloaded at: https://www.mdpi.com/article/10.3390/ijerph191912250/s1.
