# Supplementary material for: Concerted Model of Healthcare for Awá Indigenous of Nariño, Colombia
Source: Int J Environ Res Public Health. 2022 Sep 27;19(19):12250. doi: 10.3390/ijerph191912250 (PMC9566783; doi:10.3390/ijerph191912250)
Supplement: Supplementary file 1 [file ijerph-19-12250-s001.zip › APPENDIX 1. OUTLINE FOCUS GROUPS.pdf]

APPENDIX 1. OUTLINE FOR FOCUS GROUPS

This guide will be developed with three focus groups, each one formed with ten participants (pregnant women, community health agents, and traditional authorities) from the Awá indigenous reservations. The topics are directed to the analysis of the cosmovision in health of the Awá indigenous people in order to obtain elements that should be taken into account at the moment of building the health care model.

Introduction

Dear representatives of the Awá indigenous community, we cordially welcome you to the workshops convened jointly between the traditional authorities of the Awá indigenous people, the civil and health authorities of the municipality, the department and the nation with the purpose of dialoguing about the cultural elements of the health of the Awá people that should be part of a model of Health Care, based on Primary Health Care, Social Determinants and Interculturality in health. It is very important for us to listen to them, that they share with us their ideas and thoughts about their ways of organization, their health culture, about the life cycle, about the role of traditional health agents, about the uses and customs when they get sick.

We request your permission to record the conversations, take photographs, audios and videos that have the sole purpose of this research, to transcribe and capture in the documents exactly what is talked about in the meetings; neither the images nor the names will be disseminated in spaces other than academic or technical spaces proper to the process.

About the forms of organization of the Awá people

How is the Awá community organized in their indigenous communal territories, who are their traditional authorities, do they have health committees, education committees, committees that take care of the Awá territory, how are the committees conformed, what are the functions of these committees in the resguardos?

On the concept of health and illness of the Awá

What is health and what is illness for the indigenous Awá?

When an Awá indigenous person gets sick, where or to whom does he/she go for healing or attention?

Are there diseases specific to the Awá, what are they, why do they occur, how are they treated?

Do you know the diseases of the outside or white people, which ones do you know, do you know why they are produced?

What are the cares and customs that Awá women have during pregnancy?

What are the cares and customs of the Awá woman and her family during childbirth and puerperium, what does the placenta, the umbilical cord and feeding during these stages represent and what is their importance?

Do the indigenous Awá have their own health agents, what are they, and what role do they play in the prevention and cure of illnesses?

What do the indigenous Awá people get sick or die from? mention the most recent cases, especially if they are children, young people or pregnant women.

What do the indigenous Awá think about excreta and garbage, how do they dispose of this waste in their community, where do they get water for cooking, personal hygiene and housing?

On institutional health care

Have you received care at the Ricaurte Hospital for your illnesses? if you did not receive or seek care, what was the reason? if you did receive care, how did you feel? would you return to the hospital to receive care? how did you communicate and transport the patients?

What would you like the Ricaurte Hospital to have or modify in its structure, personnel or processes of attention to achieve the cure of the illnesses of the indigenous Awá that attend this health institution for their attention?

If it depended exclusively on you to build a health care model, based on Primary Health Care, Social Determinants and Interculturality in health, what would that model be like, what aspects would you modify from the current health care model, what new elements would you incorporate?

Nutritional aspects and care of indigenous Awá children

What is the daily diet of an Awá family, what food is given to newborn children, to children under 1 year old, to children from 1 to 5 years old, to older adults?

Are there attention centers - care centers for Awá children in your indigenous communal territories, such as recovery houses, what care do you give to the children in these places, who takes care of them?

Let's elaborate a map of our indigenous communal territories, where we locate the hamlets, houses, number of inhabitants, roads, schools, rivers, bridges, calculate the distances between the houses, the school, the municipal capital, describe the means of transportation used when we go to the nearest health center, the available resources and means, communications, other services and identify possible equidistant sites - strategic for the base location of the basic health teams in the Awá territory.

Organize representations, drawings, dramatizations about the meanings of the process of health, illness, about the attention that the Awá receive when they get sick and about the health situation of the Awá indigenous people.
