# Supplementary material for: Concerted Model of Healthcare for Awá Indigenous of Nariño, Colombia
Source: Int J Environ Res Public Health. 2022 Sep 27;19(19):12250. doi: 10.3390/ijerph191912250 (PMC9566783; doi:10.3390/ijerph191912250)
Supplement: Supplementary file 1 [file ijerph-19-12250-s001.zip › APPENDIX 2. OUTLINE KEY INFORMANTS.pdf]

APPENDIX 2. INTERVIEW OUTLINE FOR KEY INFORMANTS

This interview guide is directed to health sector officials from the local level of the municipality of Ricaurte, regional level of Nariño and national level (Local Health Directorate, Local Hospital of Ricaurte, Departmental Health Directorate of Nariño, Departmental University Hospital of Nariño, Ministry of Health, University of Nariño, Representatives of the Health Promotion Companies EPS, Agencies of the United Nations System and NGOs with presence in the municipality of Ricaurte).

Introduction

Welcome to these workshops convened in a concerted manner between the traditional authorities of the Awá people and the health authorities with the purpose of jointly building a model of Health Care based on Primary Health Care, Social Determinants and Interculturality in Health, aimed at the health care of the Awá indigenous people of Ricaurte Nariño Colombia.

The interview that we will conduct with each of you, explores general concepts of health, Primary Health Care, Social Determinants and Interculturality in health and inquire how they translate into the current model of health care provided in the municipality of Ricaurte to the Awá indigenous population. For us it is very important their participation and contributions according to their professional training, trajectory and work experience in the municipality.

General aspects of the concept of health, Primary Health Care and Social Determinants

What is health for you?

What is disease for you?

Do you know the concepts of Renewed Primary Health Care and Social Determinants of Health, can you explain what they refer to?

Are these concepts applied in the territorial planning for health care provided to the Awá indigenous population of the municipality of Ricaurte?

How do you imagine that the concepts, values and principles of the Renewed PHC could be translated into real actions in health care for the Awá indigenous population?

How do you imagine that the Social Determinants of Health of the Awá people could be addressed, who is responsible for addressing them, which institutions and organizations could cooperate in addressing the SDH?

Knowledge of aspects related to the health cosmovision of the Awá indigenous people and their health rights

Do you know aspects of the health cosmovision of the Awá indigenous people, which ones, what do you think about them, do you share them?

Do you know the health rights of the indigenous peoples in Colombia, what are they, are they fulfilled in the Municipality of Ricaurte?

Does the care provided by the Ricaurte Hospital to the Awá indigenous people take into account their cosmovision of health, and in what way?

Knowledge of the health situation of the indigenous Awá

Do you know or have data, studies or reports on the living conditions and health situation of the indigenous Awá, including nutritional status of children, pregnant women, older adults, coverage in health programs, epidemiological situation, demographics, basic sanitation, others, what do they show, how do you explain this health situation?

Administrative aspects, socioeconomic, geographic and cultural barriers that exist for the provision of health services to the indigenous Awá population of Ricaurte Nariño

In your institution, are there geographical, social, cultural or administrative barriers for the health care of the indigenous Awá, can you mention examples of them, how could these barriers be reduced or eliminated?

Do you believe that the current humanitarian situation caused by the armed conflict and other regional problems in the Awá indigenous communal territories would eventually prevent or limit the provision of decentralized health services in your territory?

What political, administrative, personnel, technical and financial resources conditions exist in your institution/organization that could support the implementation of a sustainable model of intercultural health care, based on Primary Health Care and Social Determinants for the Awá indigenous people?

If it were up to you to implement a health care model based on Primary Health Care, Social Determinants and Interculturality in health, how would you organize the model, what elements of the current model would you include, modify or eliminate to guarantee health care with the new model in the Awá territory?
